# The Detection of Anthrax Biomarker DPA by Ratiometric Fluorescence Probe of Carbon Quantum Dots and Europium Hybrid Material Based on Poly(ionic)- Liquid

**DOI:** 10.3390/molecules28186557

**Published:** 2023-09-11

**Authors:** Dongliang Zhang, Dongsheng Jia, Zhou Fang, Hua Min, Xiaoyi Xu, Ying Li

**Affiliations:** 1 School of Materials and Chemistry, University of Shanghai for Science and Technology, Shanghai 200093, China; zdl20003@usst.edu.cn (D.Z.); 212203158@st.usst.edu.cn (D.J.); ark2202@163.com (Z.F.); xuxy63301@163.com (X.X.); 2 Technology Transfer Center, University of Shanghai for Science and Technology, Shanghai 200093, China; 13801784422@163.com

**Keywords:** carbon quantum dots, Eu^3+^, polyionic liquid, dipicolinic acid, ratiometric fluorescence probe

## Abstract

Bacillus anthracis has gained international attention as a deadly bacterium and a potentially deadly biological warfare agent. Dipicolinic acid (DPA) is the main component of the protective layer of anthracis spores, and is also an anthrax biomarker. Therefore, it is of great significance to explore an efficient and sensitive DPA detection method. Herein, a novel ratio hybrid probe (CQDs-PIL-Eu^3+^) was prepared by a simple one-step hydrothermal method using carbon quantum dots (CQDs) as an internal reference fluorescence and a covalent bond between CQDs and Eu^3+^ by using a polyionic liquid (PIL) as a bridge molecule. The ratiometric fluorescence probe was found to have the characteristics of sensitive fluorescence visual sensing in detecting DPA. The structure and the sensing properties of CQDs-PIL-Eu^3+^ were investigated in detail. In particular, the fluorescence intensity ratio of Eu^3+^ to CQDs (I_616_/I_440_) was linear with the concentration of DPA in the range of 0–50 μM, so the detection limit of the probe was as low as 32 nm, which was far lower than the DPA dose released by the number of anthrax spores in human body (60 μM) and, thus, can achieve sensitive detection. Therefore, the ratiometric fluorescence probe in this work has the characteristics of strong anti-interference, visual sensing, and high sensitivity, which provides a very promising scheme for the realization of anthrax biomarker DPA detection.

## 1. Introduction

Anthrax is an infectious disease caused by the Bacillus anthracis, which can lead to high mortality rates upon inhalation of more than 10^4^ spores without effective medical treatment within 24–28 h [1]. Bacillus anthracis is the rod-shaped, Gram-positive, sporogenous bacterium that can cause anthracnose. Moreover, Bacillus anthracis can be ubiquitous in the soil and last for decades, even at extremely high or very low temperatures, ultraviolet radiation, and strong acid or alkaline conditions. In addition, anthrax spores are activated as soon as the surrounding environment becomes favorable. People can be infected with anthrax in a number of ways, including breathing Bacillus anthracis, inhaling anthrax-contaminated material, and touching infected animals [2]. Therefore, Bacillus anthracis is considered as a potentially lethal biological warfare agent and has been of particular concern throughout the world [3]. Hence, in order to minimize the possibility of anthrax infection and prevent bioterrorism, there is an urgent need for a widely applicable method to monitor its changes in the natural environment. Dipicolinic acid (DPA) is the main component of the protective layer of bacterial spores, accounting for 5–15% of the dry mass of the spores. Meanwhile, DPA was considered as a typical biomarker of anthrax [4,5]. Thus, it is of great significance to explore an efficient and sensitive detection method of DPA molecular.

Up to now, a variety of traditional DPA detection methods have been reported, including surface-enhanced Raman spectroscopy (SERS) [6], polymerase chain reaction (PCR) [7], high-performance liquid chromatography (HPLC) [8], gas chromatography and mass spectrometry (GC/MS) [9], electrochemical detection [10], etc. However, traditional detection methods, such as HPLC and GC/MS, require specialized operators, complex sample preparation, and expensive instruments, and immunoassay and PCR require more demanding procedures and expensive chemicals. These disadvantages may become obstacles to practical application and are not suitable for commercial use [2]. Fluorescence detection has become a more competitive choice economically due to its advantages of low cost, simple operation, high selectivity, sensitivity, fast response time, and real-time monitoring.

In recent years, rare earth fluorescent probe, especially those involving Eu^3+^ or Tb^3+^, on account of theirs unique optical and spectral properties such as long fluorescence lifetime, narrow line-like emission bands, and large stokes shift, have been considered as one of the most promising methods for DPA detection [11,12]. However, the detection of DPA based on these single-emission sensors is not reliable, because it is prone to interference from external conditions such as background light, temperature, instruments, and so on. The ratio fluorescent probe can be used for color adjustment and self-calibration by measuring the fluctuation of the ratio of two wavelengths of fluorescence intensity, so as to eliminate the interference of environment or instrument and increase the accuracy of detection results. Thus, it is considered as an ideal tool for constructing sensing platform [13,14,15]. Carbon quantum dots (CQDs), an emerging material with high photostability, good biocompatibility, low toxicity, and easy synthesis, have a wide range of applications in fluorescent probes, biological imaging, and drug delivery [16,17,18,19]. Rare earth elements also have excellent luminescence properties and have been used in a large number of applications in sensing; however, Lanthanide elements usually face problems such as poor stability and low luminescence intensity, which can be well solved by connecting rare earths with carbon quantum dots through polymers [20,21,22]. Meanwhile, by combining rare earths with carbon quantum dots, ratiometric fluorescent probes with two luminescence centers are developed to enhance the luminescence of rare earth ions through the antenna effect. At present, there is not much research on the combination of carbon quantum dots and rare earths in detecting DPA imaging, and the main research focuses on fluorescence spectroscopy detection by chelating rare earth ions on the surface of carbon quantum dots to prepare ratiometric fluorescent probes, and when DPA is added, the fluorescence intensity of carbon quantum dots does not change, while the fluorescence intensity of rare earth elements appears to change substantially as a way to achieve the detection of DPA.

Herein, we designed and developed a simple and reliable ratio fluorescent probe CQDs-PIL-Eu^3+^ for DPA detection, based on CQDs as reference fluorescence and linked to the lanthanide ion Eu^3+^ by chemical bonding of ionic liquid as a “bridge” molecule. Compared with the measurement at a single wavelength, it was found not only to have strong anti-interference, avoid the influence of external factors on the results, and achieve accurate and sensitive detection of DPA but also the detection limit of the probe was as low as 32 nM, which was far lower than the DPA dose (60 μM) released by the number of anthrax spores in human body. It has great application potential in biological and chemical analysis.

## 2. Results and Discussion

### 2.1. Structure Analysis of CQDs and CQDs-PIL-Eu^3+^

**Figure 1:**
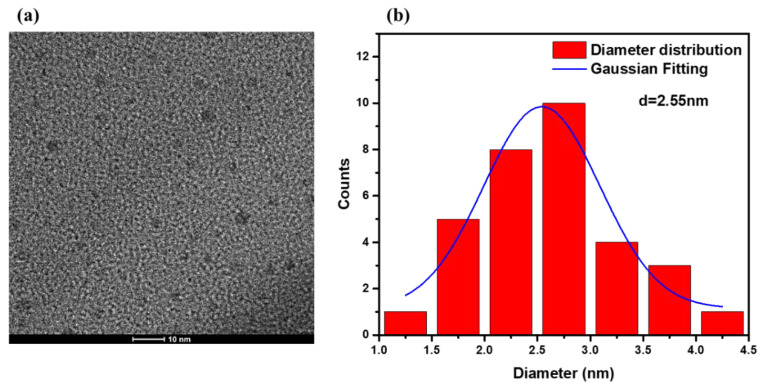
TEM characterization diagram of CQDs (**a**); Histogram of particle size distribution of CQDs (**b**).

As shown in Figure 1a, the particle size distribution of CQDs was investigated by TEM. It can be clearly seen that the prepared CQDs were regularly spherical, uniformly distributed, and had good dispersion, which indicates that the CQDs synthesized by this method [23,24]. In addition, the grain distribution histogram is given in Figure 1b, and its particle size approximately conformed to the Gaussian distribution, with an average size of about 2.55 nm.

**Figure 2:**
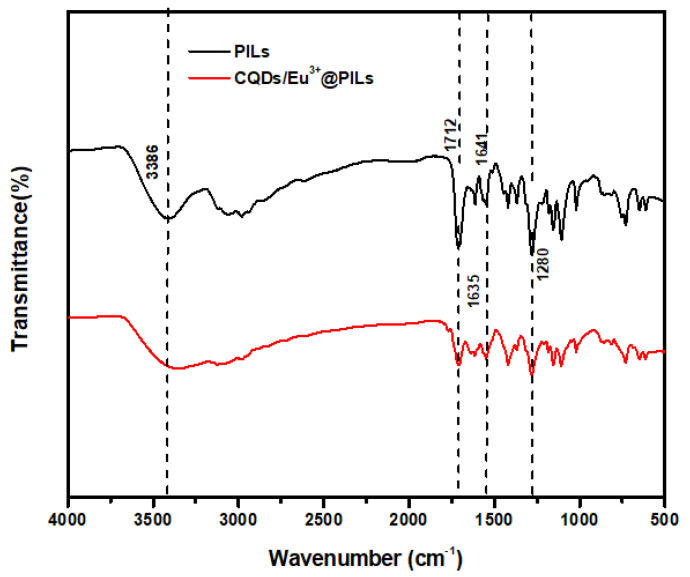
FT−IR spectra of PIL and CQDs−PIL−Eu^3+^.

To illustrate the bonding mode and surface functional groups of CQDs-PILs-Eu^3+^, the FT-IR spectra of PILs (black line) and CQDs-PILs-Eu^3+^ (red line) are given in Figure 2. The results show CQDs-PILs-Eu^3+^ with -OH, C=O, C-O and C-N stretching vibration peaks, indicating that the samples contained amino, carboxyl, and imidazole rings [25]. The characteristic vibration peak of carboxylate group appeared at 1712 cm^−1^, 1641 cm^−1^, and 1280 cm^−1^ in PILs infrared spectrum, and the stretching vibration of O-H appeared at 3386 cm^−1^, which proved the existence of carboxylate group on the surface of PILs prepared. Furthermore, the stretching vibration peak attributed to C=O shifted from 1720 cm^−1^ to 1712 cm^−1^, while the characteristic peak of the amide bond appeared at 1635 cm^−1^ in CQDs-PIL-Eu^3+^, which confirmed the successful binding of CQDs and Eu^3+^ to PIL [26,27]. In the spectrum of CQDs-PILs-Eu^3+^, the characteristic peak of amide appeared at 1635 cm^−1^, indicating that the amino group on the surface of CQDs and the carboxyl group on the surface of PILs were successfully amidezed and combined. By comparing the infrared spectra of PILs and CQDs-PILs-Eu^3+^, we found that the vibration peak of the carbonyl group was slightly blue shift, indicating that Eu^3+^ successfully chelated to the functional groups on the surface of PILs.

**Figure 3:**
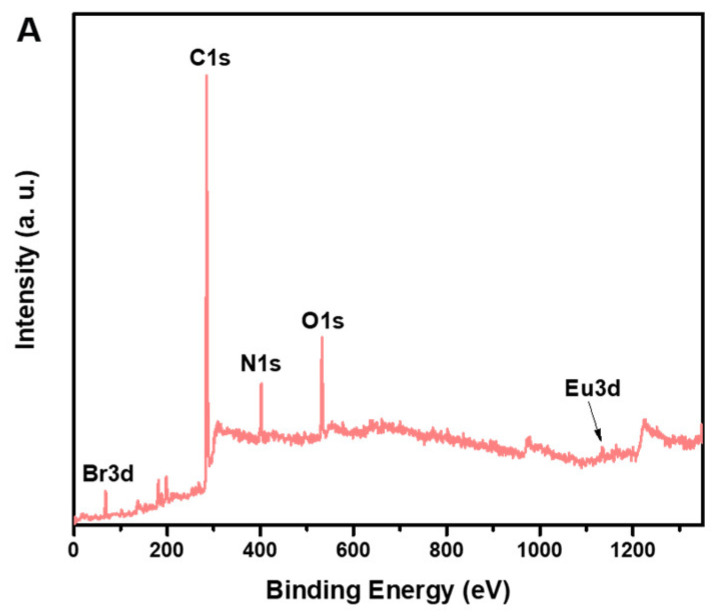

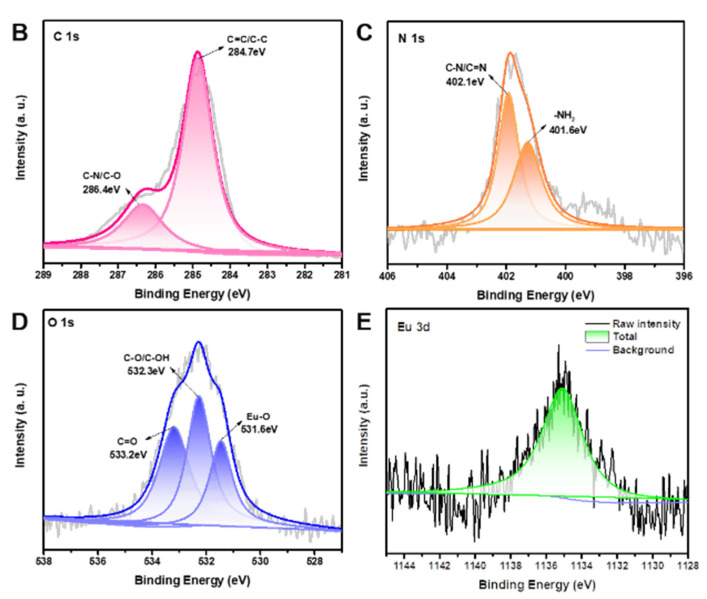
XPS spectrum of CQDs-PIL-Eu^3+^ (**A**); high-resolution XPS spectrum of C1s (**B**), N1s (**C**), O1s (**D**), and Eu3d (**E**) of CQDs-PIL-Eu^3+^.

In order to further analyze the elemental composition and functional groups of CQDs−PIL−Eu^3+^, the X-ray photoelectron spectroscopy (XPS) was performed in Figure 3A. The characteristic peaks of Br3d, C1s, N1s, O1s, and Eu3d appear at 67.83 eV, 284.81 eV, 401.75 eV, 532.3 eV, and 1134.94 eV, respectively. The results showed that the sample CQDs-PIL-Eu^3+^ was composed of bromine, carbon, nitrogen, oxygen, and europium, with the element contents of 1.88%, 79.65%, 8.9%, 8.91%, and 0.66%, respectively, which further confirmed that CQDs and Eu^3+^ were successfully bonded to PIL. In addition, high-resolution XPS spectrum revealed that C1s in CQDs-PIL-Eu^3+^ exists in the form of C-C/C=C (284.7 eV) and C-N/C-O (286.4 eV) (Figure 3B) [28], N1s in C-N/C=N (402.1 eV), and -NH_2_ (401.6 eV) (Figure 3C) [29], while O1s in C=O (533.2 eV), C-O/C-OH (532.3 eV), and O-Eu groups (531.6 eV), respectively (Figure 3D) [30]. Secondly, the fine spectra of Eu3d further affirmed the successful chelation of Eu^3+^ (Figure 3E).

### 2.2. Fluorescence Properties of CQDs and CQDs-PIL-Eu^3+^

**Figure 4:**
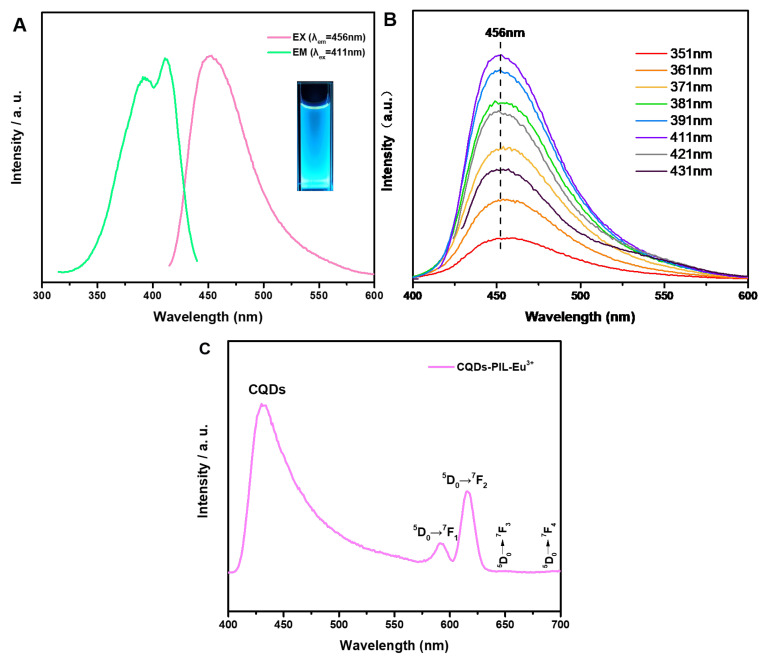
(**A**) Excitation and emission spectra of CQDs solution (illustrated with CQDs solution under 356 nm UV lamp), (**B**) fluorescence-emission spectra of CQDs solution at different excitation wavelengths, (**C**) fluorescence-emission spectra of CQDs-PIL-Eu^3+^.

The fluorescence properties of excitation and emission spectra of CQDs are shown in Figure 4A, its optimal excitation and emission wavelengths were 411 nm and 456 nm, respectively, showing characteristic emission of blue light. Meanwhile, it displayed a bright blue fluorescence (Figure 4A illustration) under a 365 nm UV lamp, and the actual results were consistent with the theory. However, in contrast to general reports, CQDs synthesized by this method showed non-excitation dependence (Figure 4B), that is, with the increase in excitation wavelength (351–431 nm), the relative fluorescence intensity of the emission spectrum was first increased and then decreased, but the location of the optimal emission peak did not change [24]. Subsequently, Figure 4C reveals that the luminescence excitation and emission spectra of CQD_S_-PIL-Eu^3+^ were studied at room temperature when excited at 298 nm. Four narrow characteristic emission spectra exhibited the 4f→4f transitions of Eu^3+^ (^5^D_0_→^7^F_J_, J = 1, 2, 3, 4), which were located at 594, 616, 653, and 695 nm, respectively. The most intense emission peak at 616 nm was ascribed to ^5^D_0_→^7^F_2_ transition, and it induced the red emissions. In addition, a characteristic emission of CQDs appeared at 440 nm, and the position of emission peak was slightly offset compared with pure CQDs, which again confirmed the successful combination of CQDs and PIL.

### 2.3. Sensing Performance of CQDs-PIL-Eu^3+^

**Figure 5:**
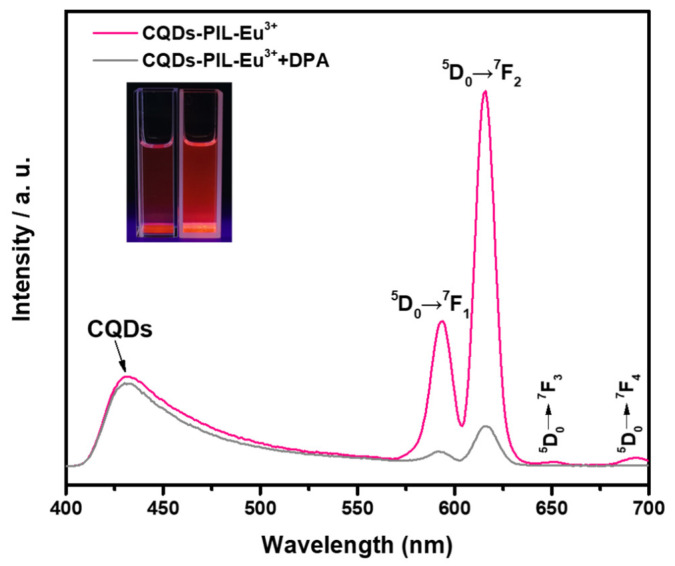
The emission spectra of CQDs–PIL–Eu^3+^ before and after dropping DPA.

The emission spectra of CQDs-PIL-Eu^3+^ in aqueous solution and its luminescence response to DPA were measured. As shown in Figure 5, the gray curve was the emission spectrum of pure solution CQDs-PIL-Eu^3+^, and the red curve was the mixed solution of CQDs-PIL-Eu^3+^ and DPA. The comparison results exhibited that the fluorescence emission intensity was significantly enhanced after the addition of 10 μM DPA, especially the intensity of the emission peak at 614 nm was increased to 9.86 times of the initial intensity. At the same time, it is easy to observe that the sample color changed from dark pink on the left to bright red on the right under the 365 nm UV lamp (inset). This not only indicates that DPA can effectively transfer energy to Eu^3+^ to sensitize its luminescence but also proves that this sample is feasible to detect DPA, and provides a simple method for naked eye recognition of DPA.

**Figure 6:**
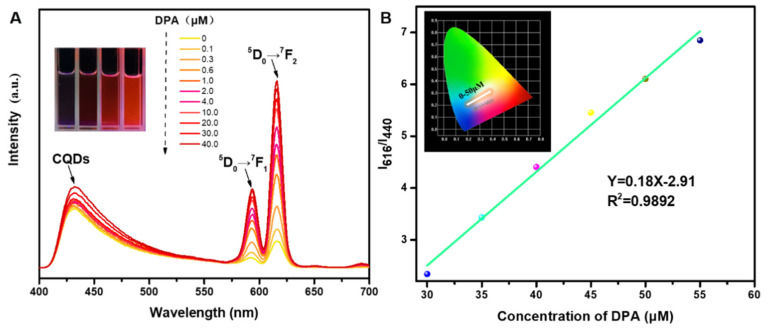
(**A**) Fluorescence emission spectra of CQDs–PIL–Eu^3+^ in DPA at different concentrations, (**B**) fluorescence response values (I_616_/I_440_) were linearly correlated with DPA concentration.

In order to further explore the quantitative recognition ability of DPA by fluorescence probe, fluorescence titration experiment was carried out at room temperature. As shown in Figure 6A, the fluorescence emission intensity of Eu^3+^ increased rapidly with the gradual addition of DPA solution, while the enhancement of CQDs was very slow and almost negligible. This indicated that DPA had a good sensitization effect on Eu^3+^, but had little effect on CQDs. Moreover, the color of the solution shifted significantly from dark pink to bright red when DPA was added and under the UV light, which can be clearly identified with the naked eye and was further affirmed by the CIE chromaticity diagram in the illustration on the right. It is noteworthy that the fluorescence intensity ratio of Eu^3+^ to CQDs (I_616_/I_440_) showed a linear relationship with the concentration of DPA in the range of 0–50 μM (Figure 6B), which conforms to the linear equation: I_616_/I_440_ = 0.18C_DPA_ − 2.91 (R^2^ = 0.9892). According to the 3σ principle, the detection limit was calculated as low as 32 nm, and the detection sensitivity was much lower than the DPA dose (60 μM) released by the number of spores causing anthrax. Compared with the lanthanide materials previously reported in Table 1, our proposed method is the best in terms of sensitivity and detection limits.

### 2.4. Possible Sensing Mechanism Analysis

**Figure 7:**
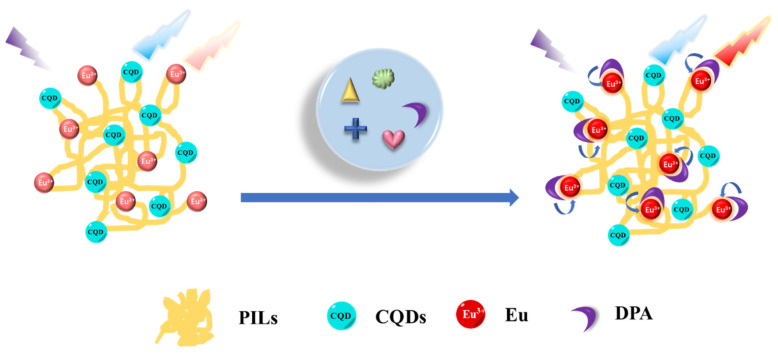
The possible mechanism of CQDs-PIL-Eu^3+^ probe recognition.

The selective recognition of DPA by CQDs–PIL–Eu^3+^ can be explained from the following two aspects: First, DPA has the structure of a three-dentate chelating ligand with one pyridine nitrogen and two carboxyl groups as coordination sites, which can have better chelating and coordination with rare earth ions compared with other aromatic ligands. Second, rare earth ions have a low efficiency of direct absorption of light energy due to the prohibitive transition, resulting in weak direct luminescence. The lowest triplet energy level of DPA is well matched with the energy required by europium ion transition, which can effectively transfer the energy through resonance coupling to Europium ion through the antenna effect, thus achieving the effect of characteristic detection (Figure 7).

## 3. Experimental Methods

### 3.1. Reagents and Apparants

N-hydroxy succinimide (NHS), anhydrous ether, absolute ethyl alcohol, and citric acid (CA) were purchased from Sinopharm Chemical Reagent Co., Ltd., (Shanghai, China). O-phenylenediamine (O-PD), 1-vinylimidazole, 4-bromomethyl benzoic acid, 1-(3-dimethylaminopropyl) -3-ethylcarbondiimide hydrochloride (EDC), poly (butylene succinate) (PBS), EuCl_3_·6H_2_O, and all the other reagents were purchased from Aladdin Chemical Regent Co., Ltd., (Shanghai, China). All chemicals are of analytical grade and were used as received without further purification.

Transmission electron microscope (TEM) measurements were conducted on a Tecanai G2 F20 S-TWIN at an accelerating voltage of 200 kV. Fourier transform infrared spectroscopy (FT-IR) in KBr pellets was obtained on a Nexus 912 AO446 spectrophotometer in the range of 4000–400 cm^−1^. X-ray photoelectric spectrometry (XPS) was performed using a Thermo Scientific K-Alpha^+^ with a monochromatic AI Kα X-ray source (1486.6 eV) operating at 12 kV and 72 W to reveal Element content and functional group types. Fluorescence spectra were measured with a RF-6000 spectrophotometer (wavelength resolution was 0.5 nm) using a xenon lamp as an excitation source.

### 3.2. Synthesis of Carbon Quantum Dots (CQDs)

Carbon quantum dots (CQDs) were prepared through a simple hydrothermal process. First, O-phenylenediamine (O-PD) (110 mg) and citric acid (CA) (0.19 g) was dissolved in deionized water (10 mL), which can be accelerated by ultrasonic dissolution. Then, the mixed solution was poured into a closed high-pressure reaction kettle with 25 mL Teflon substrate, placed in a vacuum oven, and heated at 130 °C for 5 h. After the reaction was complete, the reactor was cooled to room temperature. It was observed that the solution changed from colorless and transparent to orange-red turbid suspensions. Filtration and purification were carried out with 0.22 μm filter membrane to remove the aggregate bulk impurities. Then, the original solution was treated with the dialysis bag (MWCO 1000) for 24 h to remove small molecular impurities and obtain orange transparent CQDs aqueous solution. Finally, orange solid CQDs powder was obtained by freeze-drying.

### 3.3. Synthesis of Polyionic Liquid (PIL)

Then, 0.45 g (4.8 mmol) poly (1-vinylimidazole) and 1.24 g (5.7 mmol) 4-bromomethyl benzoic acid were dispersed in a single neck flask with anhydrous ethanol (15 mL) and sonicated. The mixture was then stirred on a magnetic stirrer and heated at 80 °C for reflux for 24 h. After cooling down, the crude product was poured into a beaker containing 30 mL anhydrous ether. Subsequently, a large number of white precipitates were precipitated, and white solids were obtained by extraction and filtration. Then, it was washed several times with absolute ether and dried for 12 h in the oven to obtain the desired product, which was imidazole-type polyionic liquid (PIL).

### 3.4. Synthesis of Ratiometric Fluorescent Probe CQDs-PIL-Eu^3+^

**Scheme 1:**
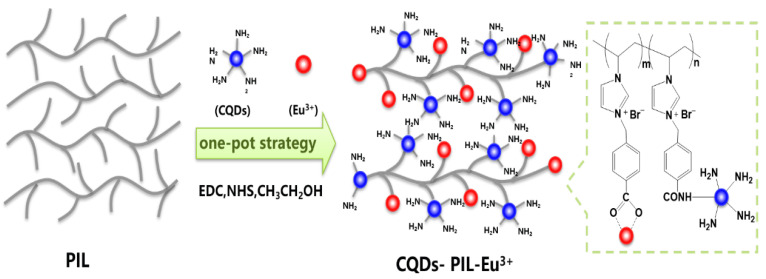
Synthesis route of CQDs-PIL-Eu^3+^.

The synthesis route is shown in Scheme 1. An amount of 0.15 g PIL was dissolved in 10 mL anhydrous ethanol in a 50 mL flask and dispersed evenly by ultrasound, then stirred on an ice bath. And 0.048 g EDC and 0.023 g NHS were added, respectively, stirred for 1 h, and removed from the ice bath. Then, 3 mg CQDs was suspended in 3 mL PBS buffer solution (pH = 7.4), and 0.092 g EuCl_3_·6H_2_O was weighed. The mixture was then added together to the flask and stirred at room temperature for 24 h. After the reaction was completed, the supernatant was removed by centrifugation, the precipitate was collected and washed with anhydrous ethanol for several times to remove the remaining impurities. The solid product CQD_S_-PIL-Eu^3+^ was finally obtained after 24 h of drying in a 65 °C oven.

### 3.5. Fluorescence Sensing Experiment

Then, 7.5 mg CQD_S_-PIL-Eu^3+^ powder was suspended in 30 mL anhydrous ethanol and dispersed evenly by ultrasonic treatment for 5 min. After that, a 0.25 mg/mL transparent suspension was formed. A pipette was used to take 3 mL of the above suspensions into a colorimetric dish, and different concentrations of DPA solutions were then added and stirred to prepare a series of parallel solutions with concentration gradients. Subsequently, the fluorescence test was carried out at the excitation wavelength of 298 nm and the width of both excitation and emission slits were all 3 nm.

## 4. Conclusions

In summary, a ratiometric fluorescence probe (CQDs-PIL-Eu^3+^) was prepared for the sensitive visual detection of DPA by a simple one-step hydrothermal method, using CQDs as the internal reference fluorescence and polyionic liquid as the “bridge” molecule to covalently connect CQDs with Eu^3+^. Obviously, the fluorescence emission intensity of Eu^3+^ increased significantly with the increase in DPA concentration, especially the emission peak intensity at 614 nm which increased to 9.86 times of the initial intensity, while the fluorescence emission intensity of CQDs increased very slowly. At the same time, it was easy to observe that the solution changed from dark pink to bright red under the 365 nm UV lamp. The above phenomenon indicates that DPA can effectively transfer energy to Eu^3+^ and sensitize its luminescence. More interestingly, the fluorescence intensity ratio of Eu^3+^ to CQDs (I_616_/I_440_) was linear with the concentration of DPA in the range of 0–50 μM, and the detection limit of the probe was as low as 32 nM. This aim of this work was to achieve accurate, sensitive, and visual-sensing detection of bacillus anthracis biomarker DPA.

## Figures and Tables

**Table 1:** Comparison of different lanthanide fluorescence probes used for DPA detection.

Lanthanide Fluorescent Probe	Detection Limit	Advantages	Reference
Magnetic Fe_3_O_4_–Tb	5.4 nM	Detection limit is extremely low	[31]
MOF–Tb	2.4 μM	Excellent anti-interference capability	[32]
SiQDs–Eu	1.02 μM	No background and self-calibration	[33]
HAP–NPs–Eu	77 nM	Detection is fast and cost is low	[34]
CQDs–PIL–Eu^3+^	32 nM	Ratio type, green non-toxic, water soluble and biocompatible, high sensitivity	In this work

## Data Availability

No new data were created or analyzed in this study. Data sharing is not applicable to this article.

## Sample Availability

Not available.
